# Induction of protein aggregation in zebrafish embryos as a method for the screening of new drugs or mutations against proteinopathies

**DOI:** 10.1016/j.mex.2018.04.004

**Published:** 2018-04-11

**Authors:** Pablo Lobos-Ruiz, Gissela Araya, Octavio Monasterio, Luis Pouchucq

**Affiliations:** 1 Laboratorio de Biología Estructural y Molecular, Departamento de Biología, Facultad de Ciencias, Universidad de Chile, Santiago, Chile; 2 Laboratorio de Biotecnología Vegetal Ambiental, Departamento de Biotecnología, Universidad Tecnológica Metropolitana, Santiago, Chile

**Keywords:** Induction of protein aggregates in zebrafish early embryos by direct microinjection of denatured protein, Intracellular protein aggregation, Zebrafish embryo, Mutant screening, Aggregate size distribution, Drug discovery, Protein aggregate induction

## Abstract

The sustained increase in the prevalence of protein aggregation related diseases requires the development of feasible methods for the design of therapeutic alternatives. The procedure traditionally used for the search of drugs or therapeutic mutations includes *in vitro* experiments, designed to prevent the aggregation of model proteins, which are then complemented with cellular toxicity studies *in vivo*, slowing down the finding of solutions. To address this, we have developed a protocol that facilitates the search of molecules and anti-aggregation mutations since it allows to evaluate their therapeutic capabilities directly in *in vivo* experiments with the use of zebrafish early embryos. Avoiding the necessity of performing *in vitro* and *in vivo* procedures separately. Giving a more realistic method for the results interpretation.

Zebrafish embryos were induced to produce intracellular aggregates of proteins by simple microinjections of known quantities of an aggregation prone protein previously labeled. The toxicity was evaluated by the survival of the embryos, while the formation of aggregates was quantified by fluorescence microscopy. The size distribution of the protein aggregates was revealed by means of ultracentrifuge sedimentation analysis.

For the development of the present method, the human γ-tubulin protein was used as model protein, which generated intracellular aggregates in more than 60% of the injected embryos. To evaluate the method, a mutation was performed that altered the state of intracellular aggregation of γ-tubulin, obtaining a significant decrease in the amount and size of the intracellular aggregates. The present method can be used for any suitable intracellular aggregation protein model.

The current method present important advantages such as:

Easy induction of intracellular aggregates.

Simple detection of intracellular protein aggregates through fluorescence microscopy and subcellular fractionation.

Overall view of the effect of drugs or mutations by combining the toxicity, the development behavior and the size distribution of intracellular protein aggregates.

## Graphical abstract

**Figure:**
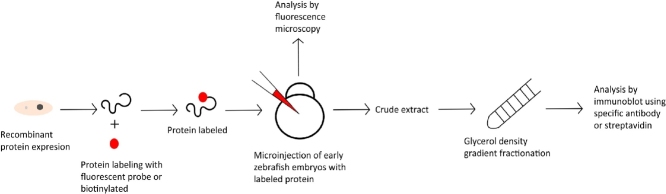

Specifications Table

**Table:** 

Subject area	Biochemistry, Genetics and Molecular Biology
More specific subject area	Protein aggregation
Method name	Induction of protein aggregates in zebrafish early embryos by direct microinjection of denatured protein.
Name and reference of original method	The in vivo novel method developed here uses microinjection of proteins into zebrafish embryos to screen drugs or mutations that interfere their aggregation.
	The original methods used *in vitro* protocols to follow protein aggregation through spectroscopy by the binding of specific probes.
	Paslawski W., Lorenzen N., Otzen D.E. (2016) Formation and Characterization of α-Synuclein Oligomers. In: Eliezer D. (eds) Protein Amyloid Aggregation. Methods in Molecular Biology, vol 1345. Humana Press, New York, NY.
Resource availability	

## Method details

### Background

The method involves the induction of zebrafish zygote-early embryo to form large intracellular aggregates induced by exogenous proteins injected into the cytoplasm by direct microinjection. The aggregation process is likely to trigger the activation of general pathways of protein misfolded response [1], including the interaction with molecular chaperones and post translational modifications. Regardless of the identity of the injected protein, or the particular way of aggregation, the intracellular aggregates can be easily detected by fluorescent microscopy. Furthermore, the toxicity can be quantified counting the survival embryos, allowing dose-effect analysis. The size distribution of the aggregates can be analyzed by cytoplasm fractionation through sedimentation in glycerol density gradient using ultracentrifugation.

## Protein preparation

### Protein purification

An aggregation-prone protein should be used as an aggregation model. The purification protocol will depend on the desired protein. Recombinant human γ-tubulin protein was used for the development of the present protocol. The protein was purified to homogeneity with purity higher than 98% (for recombinant γ-tubulin purification and aggregation refer to Pouchucq et al. [2]). Considering that γ-tubulin will naturally tend to aggregate, it is necessary to keep it denatured in solution by using denaturant buffer (8 M urea, 10 mM Na_2_S_2_O_5_, 5 mM DDT, 20 mM Tris, pH 8).

### Dialysis

A dialysis procedure is necessary in order to change the denaturant buffer for a labeling compatible buffer (8 M urea, 20mM MES, pH 7.5). The dialysis should be done against 1000 volumes of labeling buffer using a suitable dialysis membrane during one hour with permanent agitation. The labeling buffer depends on the kind of labeling method desired. Refer to the manufacturer specifications.

### Protein labeling

For this procedure, 5 mg of the denatured γ-tubulin in 1 mL of labeling buffer was conjugated with carboxytetramethylrhodamine (TAMRA®, Thermo Fischer Scientific Inc.) or Biotinylated (Biotin, Thermo Fischer Scientific Inc.) by adding the reactive probe in a 1:10 protein:probe molar ratio. The reaction was maintained on ice during one hour with recurrent agitations, and stopped by separation of the labeled protein from free probe. Urea did not interfere with the labeling reaction.

### Free probe separation

Free probe was separated through size-exclusion chromatography through Sephadex G25 (Sigma®) filtration in a columnof 20 x 0.5 cm, using the same labeling buffer as a mobile phase, at room temperature. Separation in the column of the labeled protein from the free fluorescence probe, was followed by illuminating the column with a UV lamp (UVP 254/365 nm, Upland CA, USA). The labeled protein was quantified by absorbance at 280 nm, using and extinction coefficient of 45865 for γ-tubulin. The degree of labeling was calculated following the manufacturer kit specifications.

### Labeled protein dialysis

Labeled γ-tubulin was dialyzed against 1000 volumes of injection buffer (phosphate 5 mM pH 6.8, urea 1 M) by micro-dialysis procedure using a 30 kDa cutting-off membrane. Instead of phosphate buffer, ultra-pure water could be used as the vehicle for protein microinjections. To keep the protein in solution and to avoid toxic effects on the embryo it is recommended to use the minimal concentration of urea to solubilize the protein. With recombinant γ-tubulin, 1 M urea was well tolerated by the embryos and maintained the protein soluble, avoiding protein aggregation at the moment of microinjection.

We observed that performing numerous injections with such kind of vehicle solutions did not generate undesirable effects on the embryos survival or development.

## Protein microinjection and microscopy

### Protein microinjection

Early embryos were microinjected at zygote-single-cell state [3] with 5 nanoliters (calibrated drop) of labeled γ-tubulin at 1 mg/mL, using a micro-injector (Sutter Instruments, Novato, CA.). For this procedure borosilicate needles (1.2 mm of external diameter, 0.69 mm of internal diameter and 10 centimeters long) were prepared in a puller (NARISHIGE, model PC-10) with two heat pulses at 88.3 °C and 99.2 °C.

The microinjections were performed using a micromanipulator under a magnifying glass (10x). The manual microinjection procedure is widely used in zebrafish-specialized laboratories [4]. For microinjection, several embryos can be arranged in a slotted glass or a small agar holder disposed in a Petri dish to facilitate the microinjection of hundreds of embryos in few minutes. A comprehensive manual for zebrafish microinjection can be found in Rosen et al. [5].

### Fluorescence microscopy analysis

**Fig. 1:**
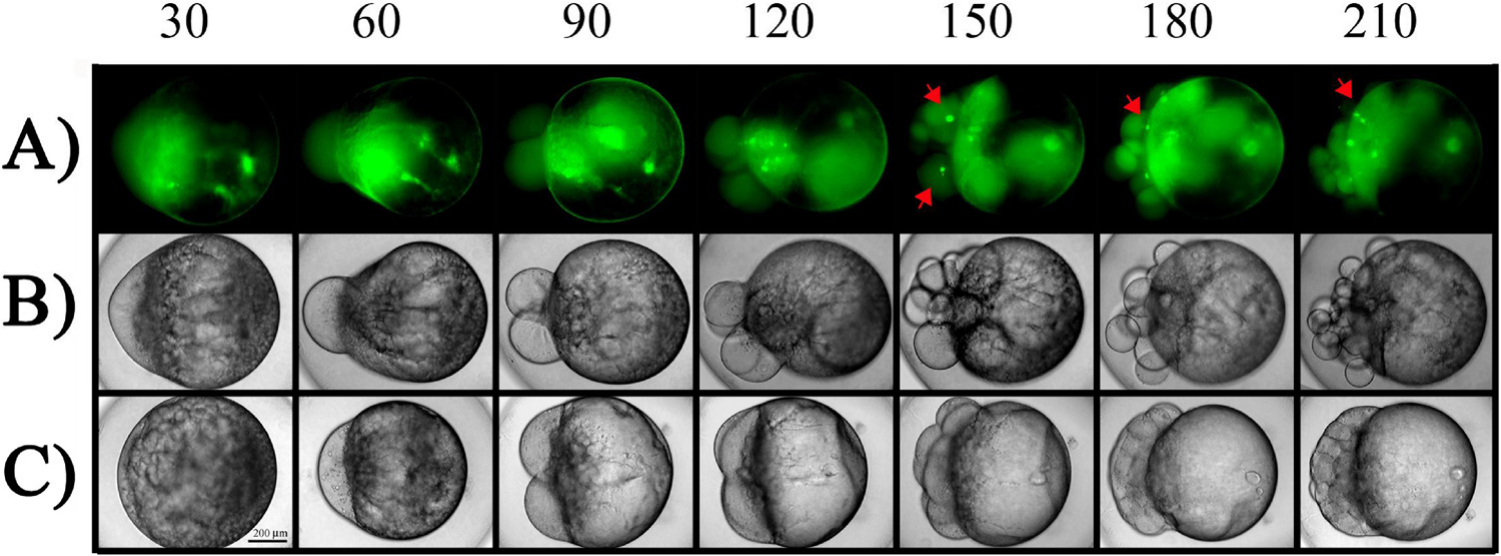
*In vivo* formation of TAMRA-labeled γ-tubulin aggregates. Fluorescence microscopy (FM) and bright field (BF) time lapse, for a representative zebrafish early embryo, microinjected with TAMRA-labeled denatured γ-tubulin, incubated at 22 °C. First row indicates post-fecundation time in minutes. A) Injected embryos observed with FM, B) the same embryo observed with BF microscopy. C) BF microscopy of the control embryo treated with vehicle solution. Red arrows depict the formation of TAMRA-labeled γ-tubulin aggregates.

The injected embryos with TAMRA-labeled γ-tubulin were mounted immediately in a compatible ELISA microplate (96-well) for observation through fluorescence microscope. The mounting media correspond to the same dish culture medium. With the usage of 10x magnification was possible to explore the whole embryo structures as it is shown in the Fig. 1. The intracellular γ-tubulin aggregate formation started after 120 minutes post fecundation. The temperature of the microscope room was maintained at 22 °C in order to ensure the proper embryos development.

### Data acquisition

**Fig. 2:**
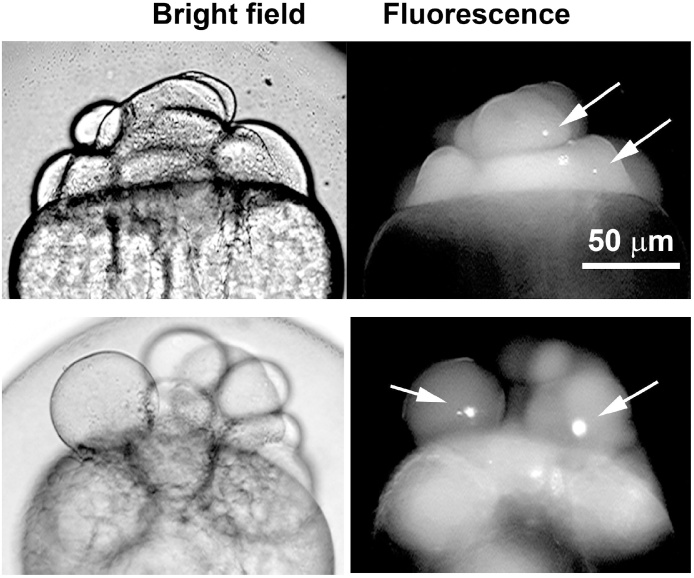
Microscopic characterization of TAMRA-labeled γ-tubulin aggregates formed *in vivo*. Bright field and fluorescence microscopy of two representative zebrafish embryos (2 hours post-fecundation) injected with TAMRA-labeled γ-tubulin. White arrows depict the formation of TAMRA-labeled γ-tubulin aggregates.

Due to some embryos died during the process, it is necessary to register the survival of them from the beginning of the experiment. Dead embryos can be registered directly by visual identification in the bright field microscope. At this point quantification of the number of aggregates formed per embryo is possible using the fluorescence microscope, as it is shown in the Fig. 2. The survival, the number of embryos forming aggregates and aggregates number per embryo data can be used to compare different treatments, for instance the anti-aggregation effect of a specific drug or mutations.

## Analysis of intracellular aggregate size distribution

1 100 biotin labeled γ-tubulin microinjected embryos were placed in a compatible ELISA microplate (96-well) and incubated for 4 hours at 28 °C, in culture medium.
2 The embryos were taken with a Pasteur pipette one by one, placed in a micro centrifuge tube and lysed in 1 ml of zebrafish lysis buffer (50 mM Hepes, pH 7.6, 1 mM EGTA, 100 mM KCl and protease inhibitor Complete®, Roche) with a homogenizer immersed in ice.
3 The lysate was cleared by centrifugation at 15,000 x g (4 °C) in a Beckman Avanti^TM^ 30 centrifuge.
4 0.5 mL of the supernatant were subjected to discontinuous glycerol density gradient prepared manually by addition of: 1 mL 5%; 1 mL 10%; 1 mL 20%; and 1 mL 40% of glycerol (in zebrafish lysis buffer) in a 5 ml ultracentrifuge tube (Beckman).
5 Ultracentrifugation was performed at 100,000 x g in a Sorvall swinging bucket AH650 rotor for 3 hours at 4 °C.
6 After ultracentrifugation, the gradients were fractionated manually from the upper zone of the gradient by taking 200 μL-volumes that were then placed in an ice-immersed micro centrifuge tube. This process can be improved by using a mechanized or automatic micro-collector.

**Fig. 3:**
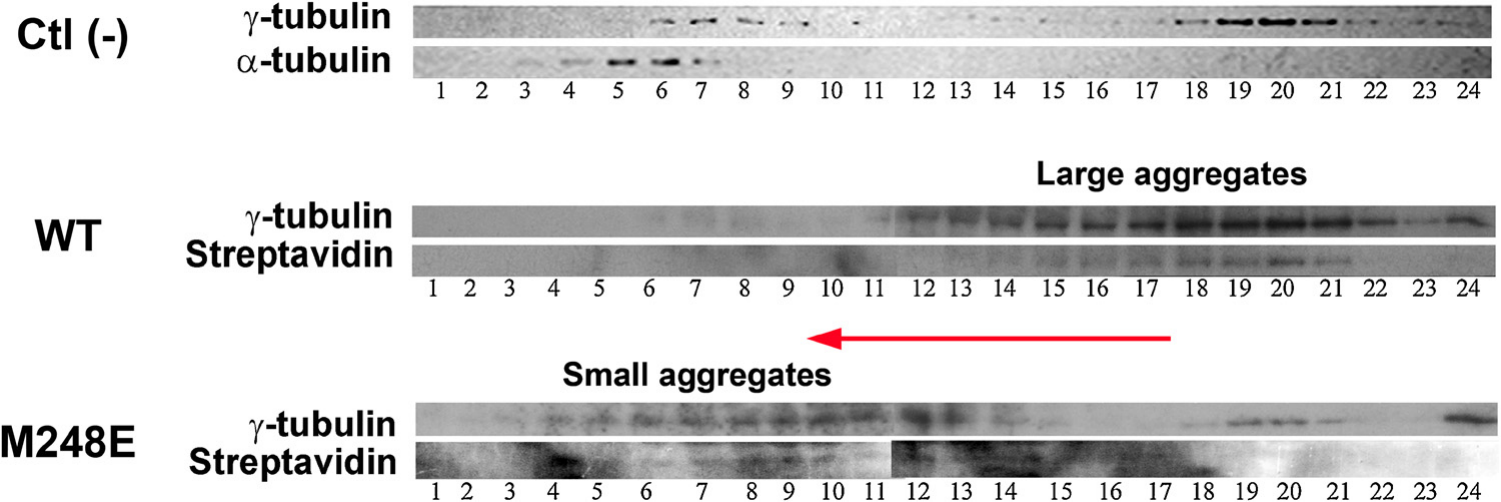
Evaluation of aggregate size distribution of endogenous γ-tubulin and exogenous biotinylated γ-tubulin. 200 nM (in the cell) of exogenous WT biotinylated γ-tubulin and biotinylated mutant γ-tubulin (ME) were microinjected in single-cell zebrafish embryos. After four hours of incubation (28 °C) the embryos were homogenized, cleared and separated by ultracentrifugation in a discontinuous glycerol density gradient (5-40%). The presence of endogenous plus exogenous γ-tubulin was detected by western-blotting (upper rows) and exogenous γ-tubulin by streptavidin (lower rows). The gradient of non-injected embryos is shown as negative control (-), α-tubulin (using Monoclonal anti-α-tubulin Clone B-5-1-2, (Sigma®) antibody) and γ-tubulin were detected by western-blotting. The red arrow indicates the sense of the shift in the distribution of the protein aggregates in the gradient due to the point mutation.

Fractions were analyzed by SDS-PAGE and western-blotting using anti-γ-tubulin monoclonal antibody (Monoclonal anti-gamma-tubulin clone GTU88, Sigma®) or streptavidin-Horseradish peroxidase conjugate (Invitrogen^TM^) (Fig. 3). Other appropriate detection methods (e.g. fluorescence) may also be used. γ-tubulin was biotinylated in order to recognize exogenous protein using the streptavidin-based detection method [6]. Application of the procedure described before is shownin Fig. 3. Previous results of our laboratory show a reduction of intracellular aggregation by the mutation M248E in γ-tubulin [2]. This residue is homologous to the one located in the loop T7 in alpha and beta-tubulin heterodimer, involved in longitudinal microtubule interactions and with the chaperonine CCT. The analysis of the gradient fractionation shown in Fig. 3 confirms a mayor change in the size distribution of the aggregates formed by the mutant protein. γ-tubulin western blotting shows that biotin has no effect over this distribution. Thus, biotin-streptavidin method confirms the identity of the exogenous protein in the aggregates.

Fluorescence microscope analysis of the embryos injected with the mutant γ-tubulin labeled with TAMRA confirmed the latest results, showing a significant decrease in embryos that formed intracellular aggregates. M248E γ-tubulin mutant induced the formation of aggregates in 13.8 ± 11.3% (n = 42) of live embryos, significantly fewer than those found for embryos injected with WT γ-tubulin (66.5 ± 8.1% (n = 53)) [2].

We suggest that this method can be applied for the discovery of new drugs applying the following experimental design: the drugs or molecules of therapeutic interest can be co-injected with the aggregation-prone protein or more simple co-incubated with the embryos in the culture medium. Effect of mutations or insertions/deletions in the aggregation pattern of the model protein can also be assessed.
